# More anterior placement of femoral tunnel position in ACL-R is associated with postoperative meniscus tears

**DOI:** 10.1186/s40634-023-00630-y

**Published:** 2023-06-30

**Authors:** Jonathan D. Hughes, Alexandra S. Gabrielli, Jonathan F. Dalton, Benjamin T. Raines, Daniel Dewald, Volker Musahl, Bryson P. Lesniak

**Affiliations:** 1 Department of Orthopaedic Surgery, University of Pittsburgh Medical Center, UPMC Freddie Fu Sports Medicine Center, 3200 S. Water St, Pittsburgh, PA 15203 USA; 2 Department of Orthopaedics, Institute of Clinical Sciences, Sahlgrenska Academy, University of Gothenburg, Gothenburg, Sweden; 3 The Hughston Clinic, Fort Walton Beach, FL USA; 4 The Hughston Foundation, Inc, Columbus, GA USA; 5 Marquette University, Milwaukee, WI USA

**Keywords:** Anterior cruciate ligament (ACL), Anterior cruciate ligament reconstruction (ACLR), Meniscus, Meniscal repair, Graft failure, Femoral tunnel position

## Abstract

**Purpose:**

The purpose of this study was to investigate the relationship between tunnel position in ACL reconstruction (ACL-R) and postoperative meniscus tears.

**Methods:**

This was a single institution, case–control study of 170 patients status-post ACL-R (2010–2019) separated into two matched groups (sex, age, BMI, graft type). Group 1—symptomatic, operative meniscus tears (both de novo and recurrent) after ACL-R. Group 2—no postoperative meniscus tears. Femoral and tibial tunnel positions were measured by 2 authors via lateral knee radiographs that were used to measure two ratios (a/t and b/h). Ratio a/t was defined as distance from the tunnel center to dorsal most subchondral contour of the lateral femoral condyle (a) divided by total sagittal diameter of the lateral condyle along Blumensaat’s line (t). The ratio b/h was defined as distance between the tunnel and Blumensaat’s line (b) divided by maximum intercondylar notch height (h). Wilcoxon sign-ranks paired test was used to compare measurements between groups (alpha set at *p* < 0.05).

**Results:**

Group 1 had average follow up of 45 months and Group 2 had average follow up of 22 months. There were no significant demographic differences between Groups 1 and 2. Group 1—a/t was 32.0% (± 10.2), which was significantly more anterior than group 2, 29.3% (± 7.3; *p* < 0.05). There was no difference in average femoral tunnel ratio b/h or tibial tunnel placement between groups.

**Conclusions:**

A relationship exists between more anterior/less anatomic femoral tunnel position and the presence of recurrent or de novo, operative meniscus tears after ACL-R. Surgeons performing ACL-R should strive for recreation of native anatomy via proper tunnel placement to maximize postoperative outcomes.

**Level of evidence:**

Level III.

## Background

The shift from isometric to anatomic anterior cruciate ligament reconstruction (ACL-R) in the last two decades is due to evidence that an anatomic strategy more closely restores native knee kinematics [12, 13, 16]. Incorrect femoral tunnel position has been reported as the most common cause of technical graft failure [8, 10, 14, 17]. A recent study demonstrated anterior femoral tunnel placement increased the risk of ACL graft failure compared to posterior femoral tunnel placement [5]. A separate study found anterior femoral and tibial tunnel placement increased the risk for graft impingement, which may be associated with decreased patient reported outcomes, graft degeneration, and re-rupture [21].

Integrity of the ACL is considered a prerequisite for meniscal repair—it has been demonstrated that insufficient reconstruction with residual rotation and antero-posterior laxity after ACL-R significantly increases the risk of subsequent de novo or recurrent meniscus tear [6, 19]. Thus, it appears that proper functioning of the ACL and the survival of the menisci are interrelated. However, a paucity of data exists regarding directly investigating the relationship between anatomic restoration of femoral and tibial tunnel positioning during ACL-R and the incidence of subsequent meniscus tears postoperatively.

The aim of the present study was to investigate whether or not more anatomic femoral or tibial tunnel position was correlated with the incidence of postoperative meniscus tears. It was hypothesized that relatively non-anatomic position of the femoral and tibial tunnels would be associated with increased incidence of subsequent symptomatic, operative meniscus tears following ACL-R. Our rationale behind this hypothetical association was the conjecture that a relatively non-anatomic positioned ACL graft may inadequately restore the protective role that the ACL plays on the menisci via antero-posterior and rotatory constraint of the tibiofemoral joint.

## Material and methods

### Inclusion/Exclusion

**Fig. 1:**
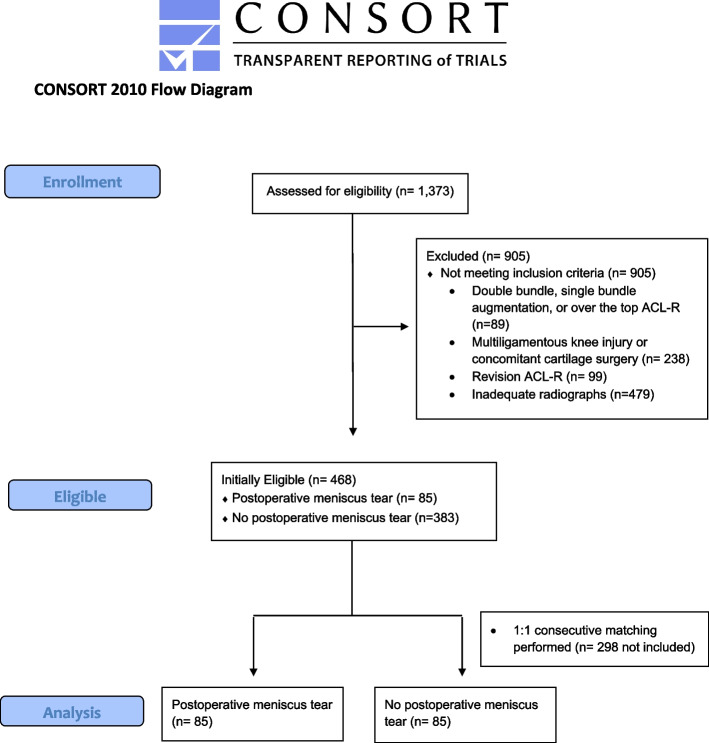
Flowchart of Inclusion and Exclusion Criteria. ACL-R: anterior cruciate ligament reconstruction

An Institutional Review Board (STUDY1903019)-approved retrospective chart review was performed using patients who had undergone ACL-R, with or without concurrent meniscus repair, between 2010 and 2019 at one institution. A search of the electronic medical record from 2010 to 2019 identified 1373 primary ACL-R procedures, which were initially reviewed for possible inclusion (Fig. 1). Inclusion criteria consisted of patients with adequate postoperative imaging, who had undergone an ACL-R with or without concurrent meniscus repair. Lateral knee radiographs were deemed adequate if they had < 6 mm of overlap between the posterior halves of the medial and lateral condyles as previously defined [18]. Patients were then excluded for inadequate imaging, double-bundle ACL-R, over the top ACL-R, single bundle ACL-R augmentation, multi-ligamentous injury, concomitant cartilage restoration surgery, or if their initial surgery constituted a revision ACL-R.

For included patients, all ACL-R surgeries were performed by one of five sports fellowship-trained orthopaedic surgeons at the same institution. All patients were initially seen in clinic and identified as having an operative ACL tear via Magnetic Resonance Imaging (MRI). MRIs were also reviewed for the presence of any concomitant meniscus injury or other ligamentous injury concurrent with ACL tear. Graft selection was based on surgeon preference after discussions with individual patients. All patients performed similar postoperative rehabilitation protocols, per the standards of practice at our institution. Patients were seen at similar intervals postoperatively. Postoperative anteroposterior (AP) and lateral x-rays of the operative knee were performed on all patients. Postoperative MRI imaging was only performed if there was concern for graft failure or other re-injury.

### Matching

A total of 468 patients met initial inclusion and exclusion criteria, of which 85 patients had postoperative meniscus tears and 383 patients did not have a postoperative meniscus tear. The 85 patients with postoperative meniscus tears were then matched to 85 patients (*N* = 170) based on age (within 3 years), sex, body mass index (BMI) (mean difference 4.4), graft type (allograft versus autograft), and preoperative tear morphology out of the remaining 383 patients in a consecutive manner. These 170 patients were separated into two groups. Group 1 was composed of patients who had a confirmed operative recurrent or de novo meniscus tear that presented during follow-up after ACL-R. MRI and subsequent arthroscopy were used to verify whether tears identified during follow-up were recurrent or de novo. Recurrent tears were defined as being of the same laterality and morphology as the original meniscus tear identified and repaired at index ACL-R. De novo tears were defined as of different laterality and/or of different morphology as the original meniscus tear identified and repaired at index ACL-R. Group 2 was composed of a matched cohort of patients who did not present after ACL-R with symptomatic meniscus tears status-post ACL-R. Demographic data as well as meniscus tear details were collected for each group. Information included age, BMI, presence/laterality/morphology of concurrent meniscus tear at time of ACL-R, and presence/laterality/morphology of recurrent or de novo tears identified during follow-up after ACL-R.

### Imaging analysis

**Fig. 2:**
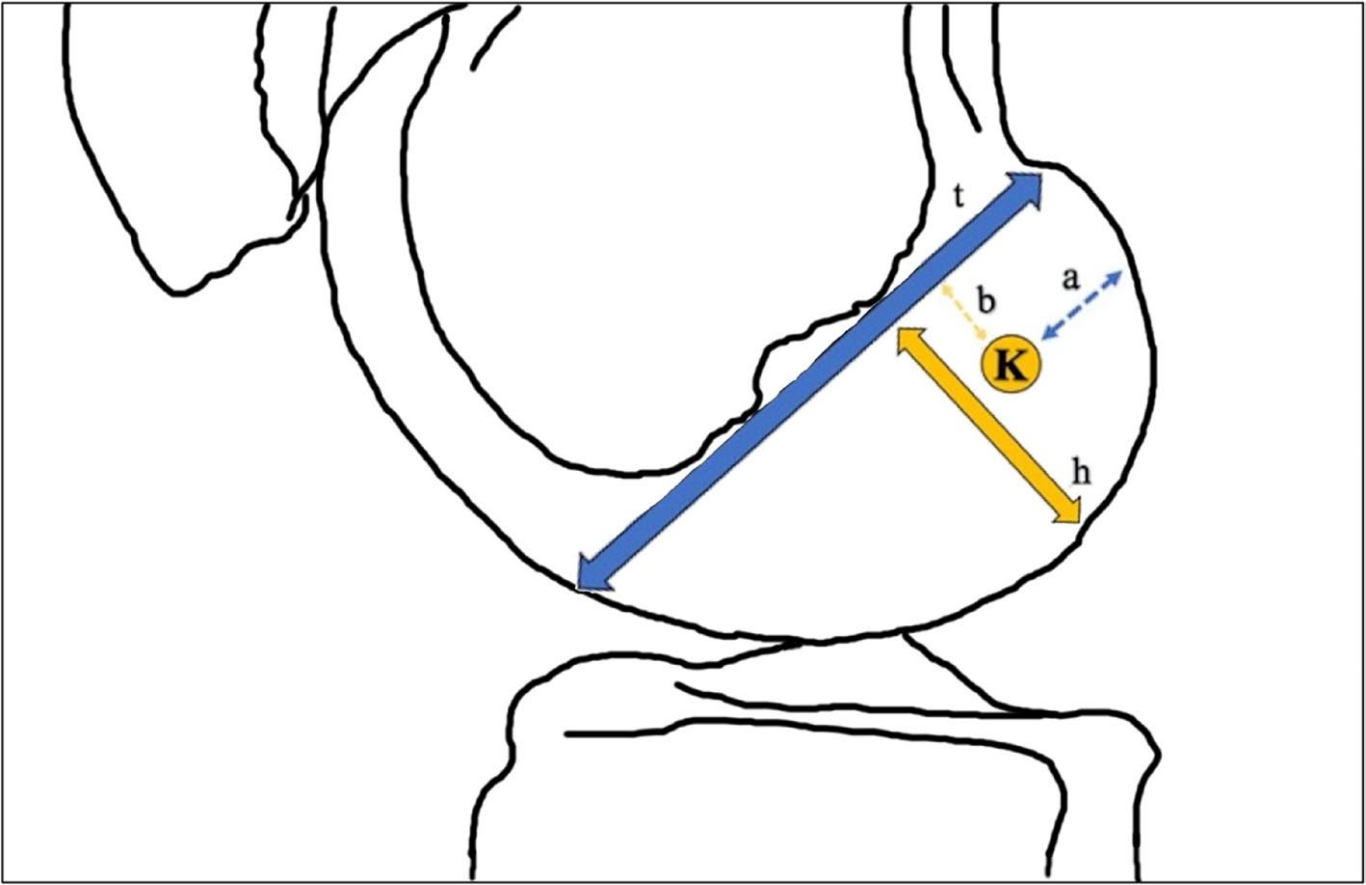
Illustration depicting the Bernard quadrant method used for radiographic measurements. Center point (**K**) of the center of the femoral tunnel. The other distances include: **t**, defined as the total sagittal diameter of the lateral condyle measured along Blumensaat’s line; **h**, the maximum intercondylar notch height, and **a**, the distance of point **K** from the most dorsal subchondral contour of the lateral femoral condyle, and **b**, which is the distance of point **K** from Blumensaat’s line

For both groups, all femoral and tibial tunnel positions were measured by two blinded reviewers using the method described by Bernard et al. [3] (Fig. 2) and Staubli et al. [22]. The measurements were performed by two fellowship-trained orthopaedic surgeons. On the femoral side, this method involves establishing a center point (k) of the femoral insertion of the ACL by measuring the anterior, posterior, proximal, and distal most borders of the femoral ACL insertion on a perfect lateral radiograph of the knee. After k is established, four distances are measured on the lateral radiograph. These distances include: t, defined as the total sagittal diameter of the lateral condyle measured along Blumensaat’s line; h, the maximum intercondylar notch height, and a, the distance of point k from the most dorsal subchondral contour of the lateral femoral condyle, and b, which is the distance of point k from Blumensaat’s line. These measurements were obtained for each patient and compared to Bernard et al.’s recommendations to assess the adequacy of anatomic positioning of the femoral ACL insertion after ACL-R. Bernard et al. report that the average anatomic position of the femoral insertion of the ACL with respect to the total sagittal diameter of the lateral femoral condyle, represented by the ratio a/t, as 24.8%, and that the average anatomic position of the femoral insertion of the ACL with respect to the notch height, represented by the ratio b/h, as 28.8% [3]. Tibial tunnel placement for each patient was determined on the same lateral radiographs by measuring the mid-sagittal tibial diameter and the center of the tibial attachment area of the ACL from the anterior tibial margin. The center of the tibial attachment was reported as a percentage of the anterior to posterior width. A prior study has determined the normal center of the anatomic footprint to be 43% the anterior to posterior width of the tibia [22].

### Statistical analysis

Continuous variables for each group were compared using Student’s t-tests. Categorical data was compared using Fisher’s test with a contingency table. A *p*-value < 0.05 was deemed statistically significant. A Wilcoxon sign-ranks paired test was used to compare post-operative femoral tunnel position measurements between groups and against averages reported by Bernard et al. [3]. Finally, an intraclass correlation coefficient (ICC) was calculated for the measurements produced by the authors using R statistics software. The inter-rater reliability (ICC: 0.87—0.88) and intra-rater reliability (ICC: 0.60—0.90) on the femoral side were found to be good to excellent. The inter-rater reliability (ICC: 0.78) and intra-rater reliability (ICC: 0.58 – 0.87) on the tibial side were also found to be good to excellent.

A sample size calculation was performed for independent study groups with continuous endpoints. For this calculation, alpha was set at 0.05 and power was set at 80%. A required sample size of 32 patients per group was required to detect a difference between the group with a diagnosed meniscus tear during follow-up and the group without a diagnosed meniscus tear during follow-up of at least one standard deviation in either dimension. Standard deviation was defined as 2.2% in the posteroanterior (PA) dimension and 2.5% in the proximal–distal (PD) dimension.

## Results

### Demographics

Group 1 and Group 2 were both composed of 85 patients each. Group 1 was demographically similar to Group 2 in terms of age, sex, average BMI, and laterality/morphology of meniscus tears at time of index ACL-R (Table 1). In contrast, Group 2 had significantly more meniscus tears at index ACL-R compared to Group 1 (Group 1: 60/85 (70.6%) patients versus Group 2: 75/85 (88%) patients with a meniscus tear at index ACL-R, *p* < 0.05). The percentage of meniscus tears repaired at the time of index ACL-R was similar between groups (Group 1: 53/60 (83%) versus Group 2: 70/75 (93.3%) meniscus tears repaired at ACL-R, n.s). Group 1 had average follow up of 45 months and Group 2 had average follow up of 24 months.

**Table 1:** Demographics table and *p*-values

		**Group 1**	**Group 2**	***p*-value**
Demographics	Age	25.7 ± 4.04	25.8 ± 4.03	n.s
	Sex	44F, 41 M		n.s
	BMI	18.1	20.0	n.s
Graft Type	Allograft	14/85 (16%)	14/85 (16%)	n.s
	Autograft	71/85 (84%)	71/85 (84%)	
Graft Material (Allograft)	Hamstring	5/14 (36%)	2/14 (14%)	n.s
	Patella bone-tendon-bone	1/14 (7%)	2/14 (14%)	
	Achilles	2/14 (14%)	1/14 (7%)	
	Anterior Tibialis	6/14 (43%)	9/14 (64%)	
Graft Material (Autograft)	Hamstring	36/71 (51%)	29/71 (41%)	n.s
	Patella bone-tendon-bone	24/71 (34%)	30/71 (42%)	
	Quadriceps	11/71 (15%)	12/71 (17%)	
Meniscus Tear Timing	Meniscus tear at time of ACL-R	60/85 (71%)	75/85 (88%)	***p* < 0.05**
	No meniscus tear at time of ACL-R	15/85 (29%)	10/85 (12%)	
	Meniscus tear repaired at time of ACL-R	53/60 (88%)	70/75 (93%)	n.s
	Recurrent or de novo tear within follow-up period	85/85 (100%)	0/85 (0%)	***p* < 0.05**
Meniscus Tear Laterality at time of ACL-R	Medial	27/60 (45%)	37/70 (53%)	n.s
	Lateral	18/60 (30%)	23/70 (33%)	
	Bilateral	15/60 (25%)	15/70 (21%)	
Meniscus Tear Morphology at time of ACL-R (medial)	Radial	9/42 (21%)	13/52 (25%)	n.s
	Bucket Handle	7/42 (17%)	12/52 (23%)	
	Oblique/Parrot Beak	2/42 (5%)	0/52 (0%)	
	Complex	0/42 (0%)	2/52 (4%)	
	Longitudinal	11/42 (26%)	11/52 (21%)	
	Horizontal	1/42 (2%)	1/52 (2%)	
	Root	1/42 (2%)	2/52 (2%)	
	Vertical	11/42 (26%)	11/52 (21%)	
Meniscus Tear Morphology at time of ACL-R (lateral)	Radial	11/33 (33%)	15/39 (38%)	n.s
	Bucket Handle	2/33 (6%)	4/39 (10%)	
	Oblique/Parrot Beak	4/33 (12%)	2/39 (5%)	
	Complex	3/33 (9%)	3/39 (8%)	
	Longitudinal	3/33 (9%)	1/39 (3%)	
	Horizontal	0/33 (0%)	1/39 (3%)	
	Root	6/33 (18%)	4/39 (10%)	
	Vertical	4/33 (12%)	9/39 (23%)	

Table detailing the demographic and perioperative differences between the patients with symptomatic, operative meniscus tears during the follow-up period status-post ACL-R (**Group 1**) and patients without symptomatic, operative meniscus tears during the follow-up period status-post ACL-R (**Group 2**). n.s: not significant, *p* > 0.05; *BMI* Body mass index, *F* Female, *M* Male, *ACL-R* Anterior cruciate ligament reconstruction

All patients in Group 1 experienced symptomatic, operative meniscus tears during follow-up (85/85, 100%). Meniscus tears identified during follow-up (Group 1) consisted of 27/85 (32%) recurrent tears, 48/85 (56%) de novo tears, and 10/85 (12%) concomitant existence of both a recurrent and a de novo tear (Table 2).

**Table 2:** Meniscus tears during follow-up period

		**Group 1**
Operative tear within follow-up period	Recurrent tear	27/85 (32%)
	De Novo tear	48/85 (56%)
	Both	10/85 (12%)

Table detailing the type (de novo versus recurrent) of meniscus tears incurred during the follow-up period status-post ACL-R (Group 1). Recurrent tears were of the same laterality and of similar morphology to tears repaired at index ACL-R. De novo tears were of different laterality or different morphology to tears repaired at index ACL-R

Graft type was similar between groups (Group 1: allograft: 14/85 (16%), autograft: 71/85 (84%) versus Group 2: allograft: 14/85 (16%), autograft: 71/85 (84%), n.s). Graft material used in patients treated with both allograft and autograft was similar between groups (Table 1).

### Femoral tunnel position

**Fig. 3:**
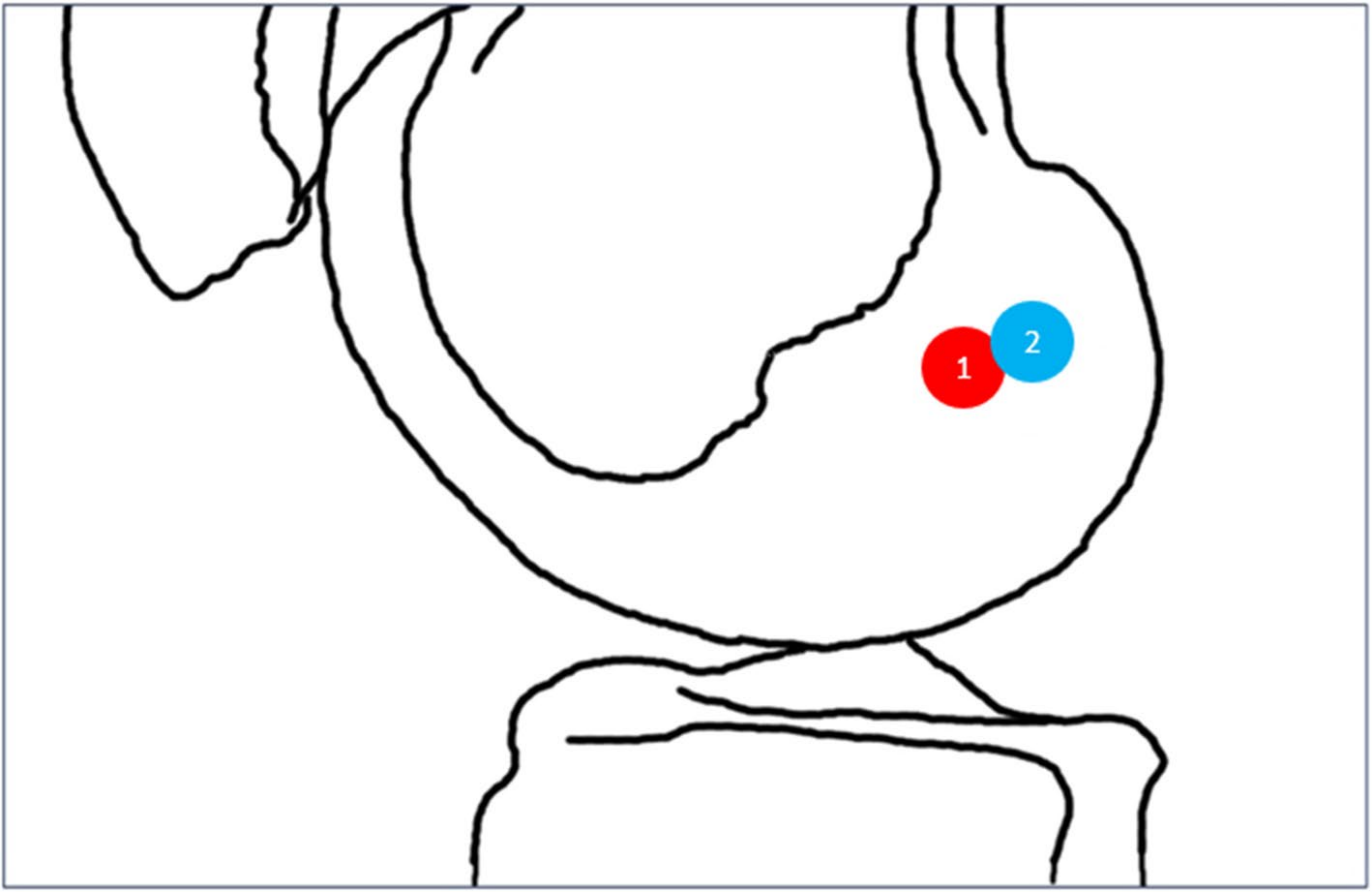
Illustration depicting the average femoral tunnel positions of Group 1 compared to Group 2

Group 1 patients had average femoral tunnel position (a/t) of 32.0% (± 10.2) and average femoral tunnel position (b/h) of 32.7% (± 10.7). In contrast, Group 2 had average femoral tunnel position (a/t) of 29.3% (± 7.3) and average femoral tunnel position (b/h) was 35.1% (± 28.3) (Fig. 3). The average (a/t) ratio for femoral tunnel position for Group 2 was significantly closer to Bernard et al.’s recommendation (*p* < 0.05). This indicates that Group 2 ACL femoral tunnels were, on average, more anatomic with respect to total sagittal diameter of the lateral femoral condyle.

### Tibial tunnel position

Group 1 patients had average tibial tunnel position of 41.5% (± 5.3) and Group 2 had average tibial tunnel position of 41.6% (± 5.7) (n.s).

## Discussion

Relatively more anterior femoral tunnel placement in ACL-R is associated with increased incidence of meniscal tears during postoperative follow-up. In contrast, patients without meniscus tears identified during follow-up, had average (a/t) ratio for femoral tunnel position that was more posterior and significantly closer to Bernard et al.’s recommendations compared to patients who experienced operative meniscus tears during follow-up. This is particularly striking given that the group of patients who did not experience a meniscus tear during follow-up had a significantly higher burden of meniscus tears identified at index ACL-R compared to patients who did experience a postoperative meniscus tear.

The ACL femoral tunnel in patients without meniscus tears identified after their index ACL-R was closer to Bernard et al.’s recommendations with respect to the total sagittal diameter of the lateral femoral condyle compared to patients who experienced an operative meniscus tear after their index ACL-R. Prior studies have emphasized the importance of anatomic femoral tunnel positioning in ACL-R in terms of restoring native knee kinematics and minimizing risk of both post-traumatic osteoarthritis (OA), ACL-R failure, and chondral and meniscus injuries [1, 5, 7, 9, 11, 15, 20]. In light of this, it is possible that patients who experienced a meniscus tear after their index ACL-R may have experienced increased rotatory knee instability secondary to a statistically significantly more vertical graft, which may have increased risk for postoperative meniscus tear. In contrast, however, the tibial tunnel position did not differ between patients with meniscus tears versus those without meniscus tears identified after their index ACL-R. This is contrary to a recent work that noted a significant association between more anterior placement of the tibial tunnel and increased anterior knee stability [4]. Further, prospective studies are warranted to evaluate this discrepancy in more detail.

The findings of this study suggest a higher risk of subsequent meniscus tears when the femoral tunnel is placed more anterior and superior to the anatomical location. Patients with meniscus tears identified during follow-up were shown to have statistically significant differences in their tunnel position in the (a/t) dimension. It is well-established that ACL rupture is a general risk factor for early development of OA and that ACL-R does not eradicate this risk [2, 20]. However, Rothrauff et al. demonstrate that anatomic ACL-R was associated with less development of posttraumatic radiographic OA when compared to non-anatomic ACL-R at long term follow up [20]. The data in our study support that non-anatomic ACL-R femoral position contributes to post-operative meniscus tears thereby potentially increasing the risk of the development of posttraumatic OA. However, it is worth noting that the difference in absolute sagittal position of the femoral tunnel was only 3% and was measured with radiographs rather than CT in patients with versus those without postoperative meniscus tears. Due to the retrospective nature of this work, it is difficult to speculate on whether or not this finding has clinical significance. CT scans are not routinely obtained postoperatively, which adds further limitations to this retrospective study. While this difference is small, it does highlight the important of precise tunnel position in order to potentially improve postoperative outcomes.

Femoral tunnel position is a known risk factor for failure of ACL-R, and has been previously cited as a cause in up to 80% of ACL-R failures [17]. Anatomic ACL-R has been described as the functional restoration of the ACL to its native dimensions, collagen orientation, and insertion sites [23]. The findings from the present study are largely in support of these previous data and demonstrate a significant inverse relationship between increasing relative anatomic restoration of the ACL femoral tunnel position with respect to the sagittal diameter of the lateral femoral condyle, and the presence of postoperative meniscus tear.

Limitations of this study include a relatively small sample size and relatively short postoperative follow-up. These factors were largely due to the matched cohort study design and the retrospective nature of this study, which also itself introduces bias. Future work to further examine the effect of femoral tunnel placement during ACL-R on postoperative meniscus tear would benefit from a prospective design. This would facilitate larger sample size with longer follow-up compared to standard postoperative care after ACL-R. Additionally, prospective work may include the use of computed tomography (CT) for tunnel measurements, rather than X-ray, which may allow for increased accuracy of tunnel measurement and increased ICC. Prospective work may also include the use of quantitative knee stability testing including KT-1000, as well as physical exam stability testing such as Lachman and pivot-shift testing in order to correlate postoperative meniscus tears with both rotatory knee instability as well as tunnel position.

## Conclusion

This study concludes that there is a relationship between the relative restoration of native anatomy during femoral tunnel creation and the presence of operative meniscus tears after ACL-R. Based on the data from this study, a more anterior femoral tunnel placement may increase the risk of operative meniscus tears after ACL-R. Therefore, anatomic femoral tunnel position is important both to preserve the reconstructed ACL and also potentially to decrease the risk of postoperative meniscus tears. Further, likely prospective, work is needed in order to both quantitatively assess rotatory knee instability and more accurately assess tunnel position via CT in patients with postoperative meniscus tears after ACL-R.

## Data Availability

The data that support the findings of this study are available from the University of Pittsburgh Medical Center but restrictions apply to the availability of these data, which were used under license for the current study, and so are not publicly available. Data are however available from the authors upon reasonable request and with permission of the University of Pittsburgh Medical Center.

## Abbreviations

ACL: Anterior cruciate ligament
ACL-R: Anterior cruciate ligament reconstruction
IRB: Institutional Review Board
MRI: Magnetic Resonance Imaging
AP: Anteroposterior
BMI: Body mass index
ICC: Intraclass correlation coefficient
PA: Posteroanterior
PD: Proximal–distal
OA: Osteoarthritis
CT: Computed tomography
M: Male
F: Female
n.s.: Not significant
